# Innovative curriculum is needed to address residents’ attitudes toward older adults: the case of geriatric trauma

**DOI:** 10.1186/s12909-022-03196-y

**Published:** 2022-02-26

**Authors:** Matthew P. Guttman, Barbara Haas, Michael Kim, Brett Mador, Avery B. Nathens, Najma Ahmed, Sarah Wheeler, Lesley Gotlib Conn

**Affiliations:** 1 Institute of Health Policy, Management, and Evaluation, University of Toronto, Toronto, Ontario Canada; 2 Department of Surgery, University of Toronto, Toronto, Ontario Canada; 3 Interdepartmental Division of Critical Care Medicine, Department of Medicine, University of Toronto, Toronto, Ontario Canada; 4 Sunnybrook Research Institute, Toronto, Ontario Canada; 5 Department of Surgery, University of Alberta, Alberta, Canada; 6 American College of Surgeons, Chicago, IL United States; 7 St. Michael’s Hospital, Unity Health, Toronto, Ontario Canada; 8 Ontario Health, Toronto, Ontario Canada

**Keywords:** Resident education, Curriculum, Geriatric, Trauma, Surgical training

## Abstract

**Background:**

Medical trainees’ negative perceptions towards older adult care have been widely reported, catalyzing targeted curricula in geriatric medicine. Little is known about surgical residents’ attitudes toward and perceptions of the educational value of caring for injured older adults. This information is needed to ensure the surgical workforce is adequately trained to care for this growing patient population. In this study, we assessed surgical trainees’ attitudes towards geriatric trauma care to inform a curriculum in geriatric trauma.

**Methods:**

We surveyed North American general surgery trainees’ beliefs and attitudes toward caring for older trauma patients, and the educational value they ascribed to learning about older trauma patient care. Descriptive statistics were used to report participant characteristics and responses.

**Results:**

Three hundred general surgery trainees from 94 post-graduate programs responded. Respondents reported too much time co-ordinating care (56%), managing non-operative patients (56%), and discharge planning (65%), all activities important to the care of older trauma patients. They recognized the importance of geriatric trauma care for their future careers (52%) but were least interested in reading about managing geriatric trauma patients (28%). When asked to rank clinical vignettes by educational value, respondents ranked the case of an older adult as least interesting (74%). As respondents progressed through their training, they reported less interest in geriatric trauma care.

**Conclusions:**

Our survey results demonstrate the generally negative attitudes and beliefs held by postgraduate surgical trainees towards the care of older adult trauma patients. Future work should focus on identifying specific changes to the postgraduate surgical curriculum which can effectively alter these attitudes and beliefs and improve the care for injured older adults.

**Supplementary Information:**

The online version contains supplementary material available at 10.1186/s12909-022-03196-y.

## Background

The dearth of residents who pursue post-graduate training in geriatric medicine despite the significant unmet need hints at the negative perceptions held by medical trainees regarding the care of older adults [1–3]. To this end, specialized geriatric curricula have been developed to modify trainee attitudes towards older adult care. Through didactic sessions and placements in geriatric day programs, rehabilitation facilities, and palliative care homes, medical schools aim to reduce aging-related bias and improve medical students’ perception of the field of geriatrics [4, 5]. Still, the vast majority of older adults will receive care by clinicians who are not geriatricians, and whose attitudes and beliefs regarding the care of older adults may impact on the quality of care provided. In surgical disciplines in particular, approaches to improving the care of older adults are urgently needed [6].

Older adults (age ≥ 65) represent a rapidly growing demographic among injured patients [7]. In North America, 40% of severely injured adults are aged over 65 [8]. While younger trauma patients often require dramatic interventions, such as massive transfusion and invasive surgery, severely injured older adults benefit from structures and processes of care using an interprofessional, interdisciplinary approach, including geriatric medicine consultation, early mobilization and multimodal analgesia [9, 10]. Currently, little is known about surgical residents’ attitudes toward and perceptions of the educational value of geriatric trauma care. This knowledge is critical in ensuring that through the appropriate curriculum and support in the trauma centre learning environment, the surgical workforce is well positioned to care for this growing population.

Our study asked how do North American general surgery residents value learning about the care of older trauma patients? Our purpose was to elicit the trainees’ attitudes and beliefs to inform a curriculum in geriatric trauma care.

## Methods

### Survey development

 Our collaborative research team began by establishing three distinct domains to be explored: (1) beliefs about older trauma patients; (2) attitudes towards caring for older trauma patients, and (3) educational value ascribed to older trauma patient care. We collected trainee demographics and measures of clinical exposure to trauma care. We iteratively generated questions to evaluate a specific domain. To avoid leading respondents with questions specific to geriatric trauma care, we framed the survey as a broad study of residents’ educational needs. We included questions not only about older adults, but also about other trauma patient groups who require specialized care including pregnant patients, burn patients, and pediatric patients.

We elected not to use existing tools, such as the Geriatrics Attitude Scale [11], which have been previously developed to assess the attitudes of medical learners toward the care of older adults. It was felt that such instruments were not specific enough to geriatric trauma patients and that their component questions were too redundant for inclusion in a concise survey.

### Survey testing & validation

We pretested individual questions in five recent graduates from our general surgery program. Testers provided written feedback to the authors on question structure, wording, and response options. Ambiguity in several questions was identified and rectified. The complete survey is available as Supplementary file. With a goal of keeping survey completion time to less than 10 min, a total of 13 questions were included in the final survey. This total included several very brief questions on respondent demographics and training stage.

After pretesting, we invited all residents in our general surgery program to complete the survey to confirm analyzability of the results. Twenty-four responses were received. As no further issues were identified, responses collected during this pilot phase were included in the final analysis.

### Study protocol

Invitation to complete the survey was sent to all general surgery training programs in the United States and all English-speaking programs in Canada. We contacted program directors and coordinators by email and asked in a cover letter to forward the invitation to all residents in their respective programs. Reminder emails were sent at two, four and nine weeks after initial dissemination. The survey was open from November 19, 2019 to January 31, 2020. Consent was implied by survey completion.

### Statistical analysis

Descriptive statistics were used to report participant characteristics and responses. Means and standard deviations (SD) or medians and interquartile ranges (IQRs) were calculated for continuous variables, as appropriate. Absolute and relative frequencies were calculated for discrete variables. All analyses were performed using SAS software (version 9.4; SAS Institute Inc., Cary, North Carolina).

 This study was approved by the Research Ethics Board at Sunnybrook Health Sciences Centre.

## Results

We collected 300 responses from 94 unique residency programs. According to data published by the Accreditation Council for Graduate Medical Education and the Canadian Residency Matching Service, there are 332 accredited general surgery programs in the United States and 14 English-speaking programs in Canada [12, 13]. Combined, these programs represent 9244 residents (8879 American, 365 Canadian). Thus, our respondents represent 3% of North American general surgery residents. However, given that our survey was distributed to residency program directors by e-mail in the hopes that they would be forwarded to residents, we are unsure of the total number of residents who received our survey invitation. The 94 unique programs from which we received at least one response represent 2679 residents. Thus, of the programs for which we are certain that our survey was distributed to residents, the response rate was 11%.

### Demographics & experience

Of 300 respondents, 54% (*n *=163) were male with an even distribution among all years of training (Table 1). Most respondents trained in American programs (81%, *n *=244) and were not considering a career as a trauma surgeon (58%, *n *=174). Respondents reported a median of two (IQR 0-4) months spent on a dedicated trauma team, four (IQR 1-8) months on a team admitting both trauma and non-trauma patients, and an additional two (IQR 1-6) months caring for trauma patients while on call.

**Table 1:** Respondent demographics & experience

	All Respondents *n* = 300
Gender, n (%)	
Male	163 (54.3)
Female	133 (44.3)
Other/Blank/Prefer not to answer	4 (1.3)
Level of training, n (%)	
PGY1	54 (18.0)
PGY2	79 (26.3)
PGY3	58 (19.3)
PGY4	52 (17.3)
PGY5+	57 (19.0)
Country, n (%)	
Canada	56 (18.7)
United States	244 (81.3)
Considering a career as a trauma surgeon, n (%)	
Yes	84 (28.0)
No	174 (58.0)
Unsure	41 (13.7)
Blank	1 (0.3)
Months spent on service, median (IQR)	
Dedicated trauma service	2 (0-4)
Service admitting both trauma AND non-trauma patients	4 (1-8)
Service caring for trauma patients on-call ONLY	2 (1-6)
Trauma laparotomies as operating surgeon or assistant, median (IQR)	6 (2-15)
Family meetings for trauma patients, participated in or been present, median (IQR)	5 (2-15)
Dedicated training in the management of geriatric trauma patients, n (%)	
ATLS	227 (75.7)
Formal faculty lead lecture	113 (37.7)
Informal teaching rounds	197 (65.7)
Other	16 (5.3)

### Trainee beliefs

**Fig. 1:**
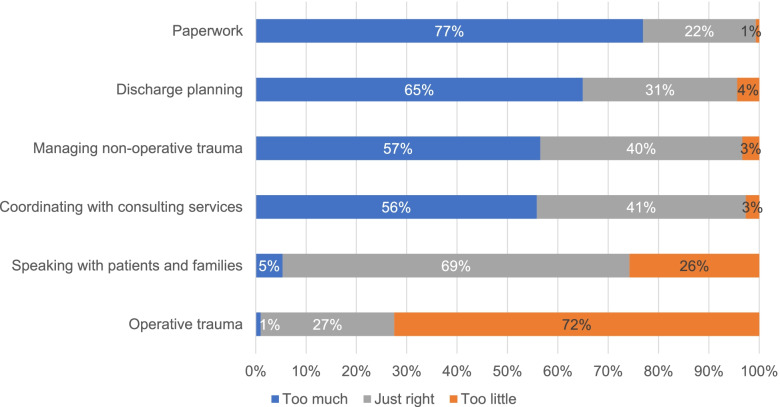
Participant responses regarding the amount of time spent on tasks while caring for trauma patients

**Fig. 2:**
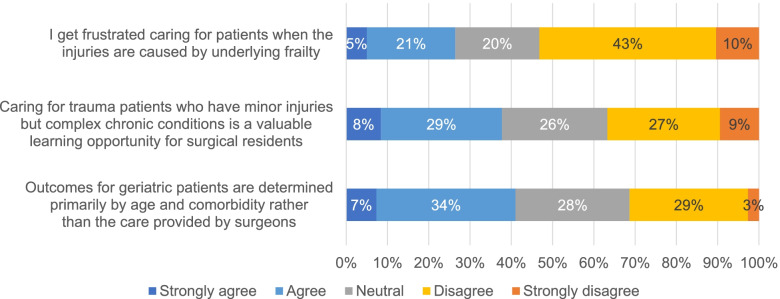
Participant responses regarding their beliefs about geriatric trauma care

When asked about caring for trauma patients, most respondents felt that they spent too much time completing paperwork (77%, *n *=230), co-ordinating with consulting services (56%, *n *=167), managing non-operative patients (56%, *n *=169), and discharge planning (65%, *n *=193) (Fig. 1). Many felt the amount of time speaking with patients and families was just right (69%, *n *=207). When asked whether “caring for trauma patients who have minor injuries but complex chronic conditions is a valuable learning opportunity for surgical residents”, respondents were evenly split between those who agreed or strongly agreed (38%, *n *=112) and those who disagreed or strongly disagreed (37%, *n *=109) (Fig. 2). Most respondents disagreed or strongly disagreed (53%, *n *=159) with feeling “frustrated caring for patients when their injuries are caused by underlying frailty.”

### Trainee attitudes

**Fig. 3:**
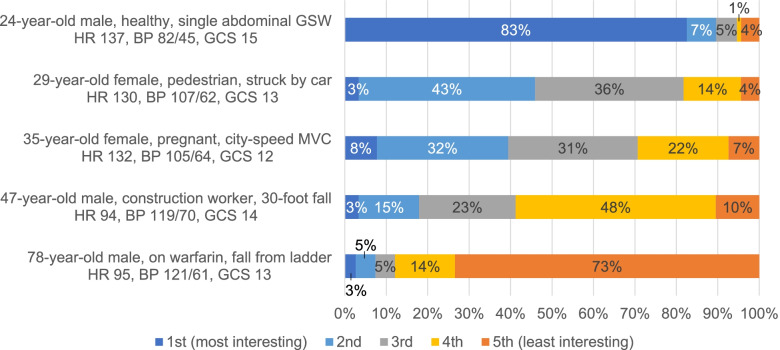
Participant responses when asked to rank clinical vignettes in terms of interest as a learner

When asked to rank clinical vignettes in terms of their interest as learners, respondents consistently ranked a vignette describing care for an older adult as the least interesting of five cases (74%, *n *=218) (Fig. 3). A middle-aged patient with comparable injury mechanism and vital signs was ranked as least interesting by only 11% (*n *=31) of respondents.

### Educational value

**Fig. 4:**
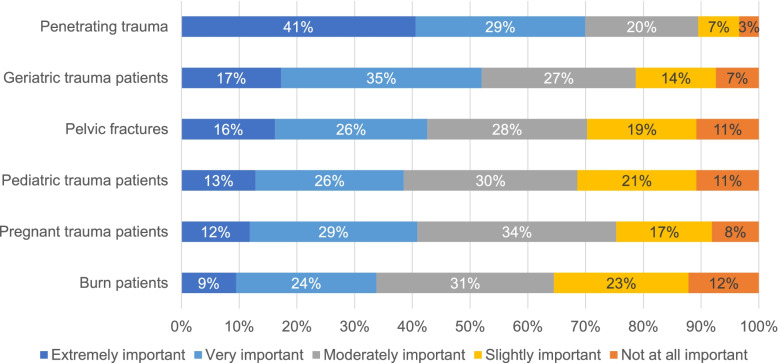
Participant responses when asked to prioritize topics within trauma care in terms of their importance for a career as a general surgeon

**Fig. 5:**
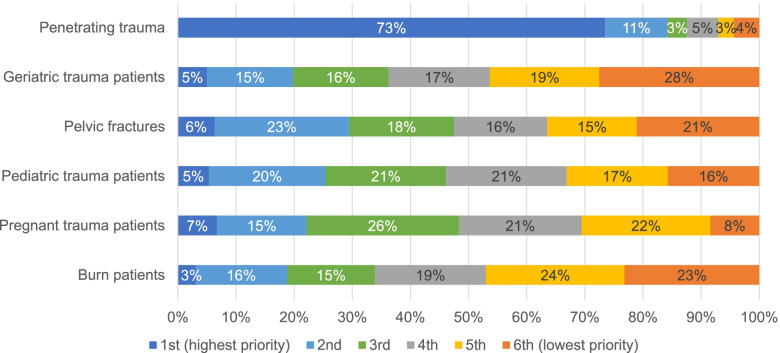
Participant responses when asked to prioritize topics within trauma care to read about

When asked to rate topics in terms of their importance for a career as a general surgeon, 52% (*n *=154) of respondents considered management of geriatric trauma patients as important or very important, second only to management of penetrating trauma (Fig. 4). Yet, when asked to prioritize reading about topics in trauma, management of geriatric trauma patients was the topic most likely to be prioritized last (28%, *n *=82) (Fig. 5).

### Effect of level of training

When stratifying responses by level of training (across each of the 5 post-graduate years), statements such as “caring for trauma patients who have minor injuries, but complex chronic conditions is a valuable learning opportunity for surgical residents” and “surgeons should routinely lead goals-of-care discussions when admitting older, frail trauma patients” were more likely to be agreed with by respondents in their early years of training. Similarly, the management of geriatric trauma patients was more likely to be viewed as important for a career in general surgery by trainees in their early years of training, and this perceived importance faded with subsequent years of training.

### Internal validity

We next evaluated responses for internal validity. Agreement with “caring for trauma patients who have minor injuries, but complex chronic conditions is a valuable learning opportunity for surgical residents” was significantly associated with greater interest in the clinical vignette involving an older trauma patient, greater perceived importance of geriatric trauma for a career in general surgery, and a higher priority placed on reading about geriatric trauma patients. Agreement with “I get frustrated caring for patients when the injuries are caused by underlying frailty” was significantly associated with lower perceived importance of geriatric trauma for a career in general surgery. The relationship between questions demonstrated either no association or an association in the direction that would be expected – the beliefs, attitudes, and perceived educational values captured by the survey appeared internally consistent among respondents.

## Discussion

Our study finds that most general surgery trainees recognize the importance of exposure to geriatric trauma care. Nonetheless, most do not find caring for older trauma patients as interesting as other trauma cases, nor do they find it as educational. Furthermore, as residents progress through their training, they report less interest engaging in and learning about geriatric trauma care.

With respect to formal post-graduate trauma curriculum, our study has important implications. The paradigm shift in medical curricula toward competency-based medical education (CBME) focuses on outcomes-based assessment of trainees. In following CBME, surgical curricula in both Canada and the US have adopted specific milestones and professional activities that trainees must acheive [14, 15]. Currently geriatric patient care has been addressed in only generic (i.e. “complex patients”) as opposed to specific terms, which may be limiting the potential for building capacity and interest in trauma care specific to older adults. A more explicit focus on achieving competence in activities essential to older adult trauma care may encourage development of relevant learning opportunities in the clinical environment and enhance perceived value of participation in geriatric trauma care. For example, the implementation of CBME provides the opportunity to assess residents on their ability to screen older trauma patients for frailty and assess for those at a high risk of functional decline. Residents could be further instructed, and assessed, on strategies to optimize transitions of care for older trauma patients and establish post-discharge supports to encourage a recovery of pre-injury function and minimize ongoing health system encounters.

In addition to shortcomings in the formal curricula, the hidden curriculum may enable negative trainee attitudes towards the care of older adults [16, 17]. As such, preceptors play a critical role in shaping their trainees’ attitudes towards older adult care and, unfortunately, many attending surgeons are found to undervalue this [1]. These attitudes may be partially due to current remuneration approaches, which reward procedure-based tasks (i.e. surgical interventions) [3, 18]. Time-intensive tasks, such as addressing goals of treatment, coordinating care, and discharge planning, are remunerated less and, therefore, valued less, despite the importance they play in ensuring favorable outcomes for older trauma patients [19].

Even with improved engagement from surgical residents, without buy-in from surgical faculty, modifying residents’ attitudes and beliefs about the educational value of these activities will prove difficult. This may require changes beyond the scope of residency training programs to ensure that care for older trauma patients is adequately valued and remunerated by health systems at large. While often delegated to members of the allied health team, tasks like discharge planning and ensuring smooth transitions of care should ultimately remain the responsibility of the attending trauma surgeon. Only through such ownership will trainees come to recognize the importance of such tasks to patient outcomes and begin to change their own attitudes and beliefs.

The trend of increasing clinical exposure leading to less positive attitudes towards older adult care has been previously described [20–22]. This phenomenon, known as empathy erosion, affects medical students and residents and it poses a potential barrier to engagement in valued learning about older adult trauma care. Empathy towards older patients is shown to improve in medical students through dedicated geriatric curricula whereby longitudinal placements with healthy older adults seem to decrease agism and improve attitudes [4, 5, 23]. Similar programs have been devised for post-graduate medical trainees; however, programs in surgical residencies have been far more limited and hampered by time and resource limitations, including resident work-hour restrictions [24–26]. Opportunities exist to design and implement innovative dedicated geriatric trauma curriculum to modify and sustain positive attitudes among senior surgical trainees.

Finally, future work will require further curricular needs assessments in order to identify interventions which can improve the beliefs and attitudes held by surgical trainees with regards to the care of older trauma patients. Such assessments should be conducted with input from relevant stakeholders. First, faculty surgeons who work with trainees should be involved in determining the domains across which further formal education is required. Second, input from interested patients could be an invaluable source of information regarding the shortcomings of surgical trainees with respect to the care of older trauma patients and the areas in which patient-centered care could be improved.

### Limitations

Our findings must be considered in the context of important limitations. First, the overall survey response rate was low. Second, we relied on individual program staff to forward our survey invitation to trainees. Nonetheless, the responses we received were well distributed across gender, PGY level, program, and country of training. The gender distribution of our respondents was consistent with that of the overall surgical resident population, as reported by the Accreditation Council for Graduate Medical Education (ACGME) [27]. Additionally, trends identified in our data, such as empathy erosion, have been well described elsewhere in the literature. Agreement between our findings and those of other authors further supports the validity of our results.

## Conclusions

As trauma systems adapt to meet the needs of injured older adults, changes in post-graduate surgical education will play an important role. These survey results demonstrate the generally negative attitudes and beliefs held by postgraduate surgical trainees towards the care of older adult trauma patients. While several hypotheses regarding the origin of these beliefs and potential mitigating strategies have been put forward, none have yet to be proven effective. Future work should focus on identifying specific changes to the postgraduate surgical curriculum which can effectively alter trainee attitudes and beliefs towards geriatric trauma care and improve the care for injured older adults.

## Supplementary Information

**Additional file 1.** Survey questions

## Data Availability

The datasets generated and/or analysed during the current study are not publicly available due to research ethics privacy regulations but are available from the corresponding author on reasonable request.

## References

[1] Bensadon BA, Teasdale TA, Odenheimer GL (2013). Attitude Adjustment: Shaping Medical Students’ Perceptions of Older Patients With a Geriatrics Curriculum. Acad Med.

[2] Institute of Medicine (US) Committee on the Future Health Care Workforce for Older Americans (2008). Retooling for an Aging America: Building the Health Care Workforce.

[3] Alliance for Aging Research (2002). Medical Never-Never Land: ten reasons why America isn’t ready for the coming age boom.

[4] Gonçalves DC (2009). From Loving Grandma to Working with Older Adults: Promoting Positive Attitudes Towards Aging. Educ Geront.

[5] Alford CL, Miles T, Palmer R, Espino D (2001). An introduction to geriatrics for first-year medical students. J Am Geriatr Soc.

[6] American College of Surgeons. Introduction to the Geriatric Surgery Verification Quality Improvement Program. 2019. United States. Available at: https://www.facs.org/quality-programs/geriatric-surgery

[7] Kozar RA, Arbabi S, Stein DM, Shackford SR, Barraco RD, Biffl WL (2015). Injury in the aged: Geriatric trauma care at the crossroads. J Trauma Acute Care Surg.

[8] Hill AD, Pinto R, Nathens AB, Fowler RA (2014). Age-related trends in severe injury hospitalization in Canada. J Trauma Acute Care Surg.

[9] Fallon WF, Rader E, Zyzanski S, Mancuso C, Martin B, Breedlove L (2006). Geriatric Outcomes Are Improved by a Geriatric Trauma Consultation Service. J Trauma Acute Care Surg.

[10] Lenartowicz M, Parkovnick M, McFarlan A, Haas B, Straus SE, Nathens AB (2012). An evaluation of a proactive geriatric trauma consultation service. Ann Surg.

[11] Reuben DB, Lee M, Davis JW, Eslami MS, Osterweil DG, Melchiore S (1998). Development and validation of a geriatrics attitudes scale for primary care residents. J Am Geriatr Soc..

[12] Accreditation Council for Graduate Medical Education (ACGME) Accreditation Data System. Available at: https://apps.acgme-i.org/ads/Public/Programs/Search

[13] Canadian Resident Matching Service (CaRMS) Program Descriptions. Canadian Resident Matching Service. Available at: https://www.carms.ca/match/r-1-main-residency-match/program-descriptions/

[14] Accreditation Council for Graduate Medical Education (ACGME) Common Program Requirements (Residency). Available at: https://www.acgme.org/globalassets/PFAssets/ProgramRequirements/CPRResidency2020.pdf. 2020. United States.

[15] The Royal College of Phyicians and Surgeons of Canada. General Surgery Competencies. 2019. Available at: https://www.royalcollege.ca

[16] Hafferty FW, Franks R (1994). The hidden curriculum, ethics teaching, and the structure of medical education. Acad Med.

[17] Stall N (2012). Time to end ageism in medical education. CMAJ.

[18] Golden AG, Silverman MA, Mintzer MJ (2012). Is Geriatric Medicine Terminally Ill?. Ann Intern Med.

[19] Farber J, Siu A, Bloom P (2007). How much time do physicians spend providing care outside of office visits?. Ann Intern Med.

[20] Kishimoto M, Nagoshi M, Williams S, Masaki KH, Lanioe Blanchette P (2005). Knowledge and Attitudes About Geriatrics of Medical Students, Internal Medicine Residents, and Geriatric Medicine Fellows. J Am Geriatr Soc.

[21] Newton BW, Barber L, Clardy J, Cleveland E, O’Sullivan P (2008). Is There Hardening of the Heart During Medical School?. Acad Med.

[22] Bellini LM, Shea JA (2005). Mood change and empathy decline persist during three years of internal medicine training. Acad Med.

[23] Maurer MS, Costley AW, Miller PA, McCabe S, Dubin S, Cheng H (2006). The Columbia Cooperative Aging Program: an interdisciplinary and interdepartmental approach to geriatric education for medical interns. J Am Geriatr Soc.

[24] Prendergast HM, Jurivich D, Edison M, Bradshaw Bunney E, Williams J, Schlichting A (2010). Preparing the Front Line for the Increase in the Aging Population: Geriatric Curriculum Development for an Emergency Medicine Residency Program. J Emerg Med.

[25] Barbas AS, Haney JC, Henry BV, Heflin MT, Lagoo SA (2014). Development and implementation of a formalized geriatric surgery curriculum for general surgery residents. Gerontol Geriatr Educ.

[26] Potter JF, Burton JR, Drach GW, Eisner J, Lundeberg NE, Solomon DH (2005). Geriatrics for Residents in the Surgical and Medical Specialties: Implementation of Curricula and Training Experiences. J Am Geriatr Soc.

[27] Accreditation Council for Graduate Medical Education (ACGME) data resource book. Available at https://acgme.org/about-us/publications-and-resources/graduate-medical-education-data-resource-book/.

