# Supplementary material for: Innovative curriculum is needed to address residents’ attitudes toward older adults: the case of geriatric trauma
Source: BMC Med Educ. 2022 Feb 26;22:130. doi: 10.1186/s12909-022-03196-y (PMC8881881; doi:10.1186/s12909-022-03196-y)
Supplement: Supplementary file 1 — Additional file 1. Survey questions [file 12909_2022_3196_MOESM1_ESM.docx]

**Supplemental File:** Survey questions

1. **Demographics**
2. What is your gender? (Male/Female/Prefer not to answer/Other)
3. Please indicate your current level of clinical training: (If currently on a research year, please indicate the last year of clinical training completed) (PGY1/2/3/4/5/Prefer not to answer/Other)
4. Are you considering a career as a trauma surgeon? (Yes/No/Unsure)
5. **Clinical & training experiences**
6. Up to this point in your residency, approximately how many months have you spent: (Leave blank if unsure or can't recall)
   1. As part of a dedicated trauma team/service (admitting ONLY trauma patients)
   2. As a member of a surgical team/service that admits both trauma AND non-trauma patients
   3. Caring for trauma patients on-call while not assigned to a trauma team/service during regular duty hours
7. Approximately how many trauma laparotomies have you scrubbed in for either as the operating surgeon or as an assistant over your entire residency training?
8. Approximately how many family meetings regarding goals of care or end of life care have you participated in or been present for while caring for trauma patients over your entire residency training?
9. Have you had any dedicated/specialized training regarding the management of injuries in the following patient populations: (ATLS, Formal faculty lead lecture, Informal teaching rounds (e.g. on ward, in trauma bay, in OR,  during morning report), Other; select all that apply)
   1. Geriatric trauma patients
   2. Pregnant trauma patients
   3. Pediatric trauma patients
   4. Burn patients

If you selected “other”, please specify: (free text)

1. **Beliefs**
2. How would you rate the amount of time you spent on the following tasks while caring for trauma patients: (too little, just right, too much)
   1. Operative trauma
   2. Paperwork
   3. Co-ordinating with consulting services
   4. Talking with patients and their families
   5. Managing non-operative trauma patients
   6. Discharge planning
3. Please indicate your level of agreement with the following statements: (strongly agree, agree, neutral, disagree, strongly disagree)
   1. Outcomes for geriatric patients are determined primarily by age and comorbidity rather than the care provided by surgeons
   2. Outcomes for burn patients are determined primarily by age and comorbidity rather than the care provided by surgeons
   3. Much greater effort is required to bring geriatric patients back to their previous state of health than younger patients.
   4. Caring for trauma patients who have minor injuries but complex chronic conditions is a valuable learning opportunity for surgical residents.
   5. Surgeons should routinely lead goals-of-care discussions when admitting older, frail trauma patients
   6. Geriatricians should routinely lead goals-of-care discussions when admitting older, frail trauma patients
   7. I get frustrated caring for patients when the injuries are caused by underlying psychiatric problems
   8. I get frustrated caring for patients when the injuries are caused by underlying frailty
   9. As a surgical resident I am uncomfortable with the increased legal risk when caring for pregnant trauma patients.
4. **Attitudes**
5. Rank the following patients in terms of how interesting you would find them as a learner:
   1. 24-year-old male, previously healthy, single abdominal GSW, HR 137, BP 82/45, GCS 15
   2. 78-year-old male, on warfarin, fall from ladder, HR 95, BP 121/61, GCS 13
   3. 29-year-old female, pedestrian, struck by car, HR 130, BP 107/62, GCS 13
   4. 35-year-old female, pregnant, city-speed MVC, HR 132, BP 105/64, GCS 12
   5. 47-year-old male, construction worker, 30-foot fall, HR 94, BP 119/70, GCS 14
6. **Educational value**
7. Rate the following topics in terms of their importance for your future career as a general surgeon: (not at all important, slightly important, moderately important, very important, extremely important)
   1. Management of penetrating trauma
   2. Management of geriatric trauma patients
   3. Management of pelvic fractures
   4. Management of pregnant trauma patients
   5. Management of burn patients
   6. Management of pediatric trauma patients
8. If you had 60 minutes to read about a topic in trauma, how would you prioritize the following:
   1. Management of penetrating trauma
   2. Management of geriatric trauma patients
   3. Management of pelvic fractures
   4. Management of pregnant trauma patients
   5. Management of burn patients
   6. Management of pediatric trauma patients
9. Which factor was most important in deciding which topic to prioritize above? (choose 1)
   1. What I felt was most interesting
   2. What I felt was most important to my future practice as a general surgeon
   3. Where I felt my knowledge was most lacking
   4. Other: (free text)
